# Neurological Immune-Related Adverse Events Induced by Immune Checkpoint Inhibitors

**DOI:** 10.3390/biomedicines12061319

**Published:** 2024-06-13

**Authors:** Sotiria Stavropoulou De Lorenzo, Athina Andravizou, Harry Alexopoulos, Iliana Michailidou, Alexandros Bokas, Evangelia Kesidou, Marina-Kleopatra Boziki, Dimitrios Parissis, Christos Bakirtzis, Nikolaos Grigoriadis

**Affiliations:** 1 Second Department of Neurology, School of Medicine, Aristotle University of Thessaloniki, 54621 Thessaloniki, Greece; iradel7714@gmail.com (S.S.D.L.); athina.andravizou9@hotmail.com (A.A.); ilianam@auth.gr (I.M.); kesidoue@auth.gr (E.K.); bozikim@auth.gr (M.-K.B.); dparisis@auth.gr (D.P.); ngrigoriadis@auth.gr (N.G.); 2 Department of Cell Biology and Biophysics, Faculty of Biology, National and Kapodistrian University of Athens, University Campus, 15784 Athens, Greece; halexo@gmail.com; 3 Department of Medical Oncology, Theageneio Cancer Hospital, 54639 Thessaloniki, Greece; alexanderbokas@outlook.com

**Keywords:** immune checkpoint inhibitors, neurological immune-related adverse events, neurotoxicity, paraneoplastic neurological syndromes, antineuronal antibodies, central nervous system, peripheral nervous system

## Abstract

The use of immune checkpoint inhibitors (ICIs) for the treatment of various advanced and aggressive types of malignancy has significantly increased both survival and long-term remission rates. ICIs block crucial inhibitory pathways of the immune system, in order to trigger an aggravated immune response against the tumor. However, this enhanced immune activation leads to the development of numerous immune-related adverse events (irAEs), which may affect any system. Although severe neurological irAEs are relatively rare, they carry a high disability burden, and they can be potentially life-threatening. Therefore, clinicians must be alert and act promptly when individuals receiving ICIs present with new-onset neurological symptoms. In this narrative review, we have collected all the currently available data regarding the epidemiology, pathogenesis, clinical manifestations, diagnosis, and treatment of post-ICI neurological irAEs. This review aims to raise physicians’ awareness, enrich their knowledge regarding disease pathogenesis, and guide them through the diagnosis and management of post-ICI neurological irAEs.

## 1. Introduction

The introduction of immunotherapy in cancer treatment strategies with potent immune checkpoint inhibitors (ICIs) has revolutionized cancer therapy. ICIs are monoclonal antibodies targeting specific regulatory proteins, involved in inhibitory pathways of the immune system. The inhibition of these immune regulators, known as immune checkpoints (ICPs), unleashes an aggravated immune response against the tumor [1,2].

Tumors employ various mechanisms through which they manage to escape immune surveillance and decrease the effectiveness of the immune system, in order to ensure their survival and uncontrolled proliferation [3,4]. Furthermore, mutational processes are highly implicated in the mechanism of oncogenesis. These mutations give rise to new tumor antigens, known as neo-antigens, which enrich tumor genetic diversity and consequently increase tumor resistance against certain cancer therapies. The awakening and reinforcement of the immune system to attack underlying malignancies, regardless of their mutational load, has been achieved with ICIs [5].

Currently, four main categories of ICIs have been approved by the Food and Drug Administration (FDA), targeting cytotoxic T lymphocyte-associated antigen 4 (CTLA-4), programmed death 1 (PD-1) receptor, programmed death ligand 1 (PD-L1), and lymphocyte activation gene 3 (LAG-3) [6]. Ipilimumab, targeting CTLA-4, was the first ICI approved for the treatment of metastatic melanoma, in 2011 [7]. To date, 14 ICIs are available for the treatment of various malignancies, including renal cell carcinoma, non-small cell lung cancer (NSCLC), small cell lung cancer (SCLC), and advanced Hodgkin’s lymphoma among other tumors [6,8]. Additionally, numerous ongoing clinical trials are currently evaluating the safety and efficacy of novel therapeutic agents targeting both inhibitory and stimulatory ICPs, including the inducible T cell co-stimulator (ICOS), T cell Ig and ITIM domain (TIGIT), B cell and T lymphocyte attenuator (BTLA), T cell immunoglobulin and mucin domain containing 3 (TIM-3), and V-domain Ig-containing suppressor of T cell activation (VISTA) [8]. ICIs are being widely used for the treatment of advanced malignant diseases and, according to studies, they have significantly increased both survival and long-term remission rates [6,7].

ICIs succeed in fighting advanced and aggressive types of malignancies, by fueling immune system responses at the cost of a wide spectrum of immune-related adverse events (irAEs), which might affect any organ or system, frequently mimicking classic autoimmune disorders [6]. About 90% of the individuals treated with ICIs have reported the occurrence of irAEs. The most commonly encountered irAEs involve the skin, gastrointestinal tract (GIT), and endocrine system, leading to the development of dermatitis, colitis, hepatitis, thyroiditis, and hypophysitis [5]. Although the vast majority of studies report the incidence of irAEs altogether, according to the category of ICI administered and not the type of malignancy, a few studies categorized irAEs caused by the administration of any ICI based on the type of malignancy. Interestingly, a tumor-specific pattern of irAEs has been revealed [9,10,11]. In particular, individuals receiving ICIs for the treatment of lung cancer (both SCLC and NSCLC) have an increased risk of developing pneumonitis, whereas those with colorectal carcinoma usually present with colitis [11,12,13]. Surprisingly, the administration of ICIs for the treatment of different hematological malignancies gives rise to distinctive irAEs in each type of malignancy; although skin-related irAEs were reported in both groups, individuals with acute myeloid leukemia (AML) may develop transaminitis, whereas those with Hodgkin’s lymphoma usually present with colitis [11]. Despite the increased incidence of irAEs, the vast majority of these are reversible. A grading system of irAEs has been developed by the Common Terminology Criteria for Adverse Events (CTCAE), which categorizes irAEs according to their clinical manifestations and outcomes. Specifically, grade 3, grade 4, and grade 5 irAEs are classified as severe, life-threatening, and fatal, respectively [14]. The prevalence of severe irAEs with CTLA-4 inhibitors was estimated at 15–42%, whereas more recently developed ICIs have been associated with lower numbers. However, ICI combination treatment strategies with CTLA-4 and PD-1/PD-L1 agents appear to give rise to severe (grade ≥ 3) irAEs in 40–45% of patients [14,15].

Neurological irAEs are relatively uncommon, and they are usually either mild or moderate, such as headache and peripheral neuropathy. Severe (grade ≥ 3) neurological irAEs rarely occur in individuals being treated with ICIs (<1%) [16]. However, they are of utmost importance since they are disabling and may be potentially life-threatening or even fatal [17,18]. Neurological irAEs may affect any part of the neuroaxis, including the central nervous system (CNS), peripheral nervous system (PNS), neuromuscular junction, and muscle. Often, they are multifocal and may appear concomitantly with other irAEs. Out of these, neurological irAEs affecting the neuromuscular junction are the most prevalent [10,11]. ICIs enhance the immune system and boost the immune response, so that the immune system recognizes and effectively destroys tumor cells. Increased T cell activation against tumor antigens, with concomitant epitope spreading caused by accelerated tumor lysis, leads to the formation of primed cytotoxic T cells, as well as antibodies against tumor antigens [5]. Several times tumors may express antigens (aberrant expression), which are physiologically expressed by the nervous tissue [6]. Therefore, an aggravated immune response against tumor antigens may also lead to the development of neurological irAEs [6,16]. It is important to mention that ICIs may also give rise to paraneoplastic antineuronal antibodies and manifestations closely resembling paraneoplastic neurological syndromes [6]. This observation was first described in patients receiving ICIs for the treatment of metastatic melanoma, although melanoma had not been associated with paraneoplastic neurological syndromes, until then [19]. Similar results were seen in individuals being treated with ICIs for renal cell carcinoma, another malignancy previously unrelated to the development of paraneoplastic neurological syndromes [20]. Currently, the overall incidence of paraneoplastic neurological syndromes has significantly increased with the widespread administration of ICIs [6]. The wide spectrum of neurological irAEs may be partially explained by the mechanism of action of ICIs.

## 2. Materials and Methods

The present article reviews the currently available literature regarding neurological irAEs caused by the administration of ICIs, including their epidemiology, pathogenesis, clinical manifestations, diagnosis, management, and outcomes. For the conduct of this review, the databases PubMed, Medline, and Scopus were accessed. The terms “immune checkpoint inhibitors”, “neurotoxicity”, “neurological immune-related adverse events”, “paraneoplastic neurological syndromes”, “antineuronal antibodies”, “central nervous system”, “peripheral nervous system” and “treatment” were used to yield results. For the selection of our material, we only collected articles written in English and published within the last decade, including reviews, clinical studies, animal studies, and meta-analyses. Articles written in a language other than English or with older dates of publication were excluded. Finally, we carefully evaluated all related articles and combined the valuable information to synthesize this current review.

## 3. Mechanism of Action of ICIs

Each main category of ICIs targets one ICP: CTLA-4, PD-1, PD-L1, or LAG-3. Focusing on tumor microenvironment (TME), CTLA-4, PD-1, and LAG-3 are expressed by immune cells, whereas PD-L1 is expressed by tumor cells. CTLA-4, PD-1, and LAG-3 are negative immune regulators, limiting immune responses upon stimulation [5,8,21].

**Figure 1:**
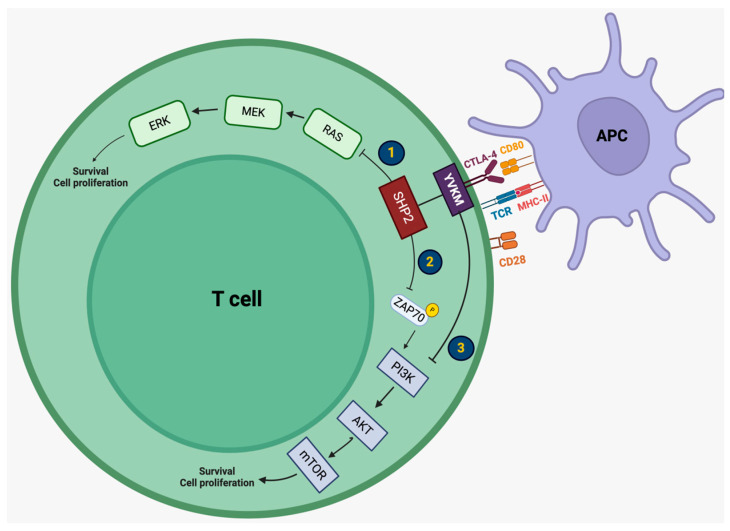
Intracellular mechanisms of CTLA-4 action in T lymphocytes. Activation of CTLA-4 inhibits T cell survival via three proposed mechanisms: (1) the inhibition of the RAS/MEK/ERK1/2 pathway by SHP2, (2) the inhibition of ZAP70 phosphorylation by SHP2, blocking the PI3K/AKT/mTOR pathway, (3) the direct inhibition of the PI3K/AKT/mTOR pathway by the YVKM motif. CTLA-4: cytotoxic T lymphocyte-associated antigen 4, CD80: cluster of differentiation 80, TCR: T cell receptor, MHC-II: major histocompatibility complex-II, CD28: cluster of differentiation 28, SHP2: Src homology region 2-containing protein tyrosine phosphatase 2, ZAP70: zeta-chain-associated protein kinase 70, PI3K: phosphoinositide 3-kinase, AKT: protein kinase B (PKB, also known as AKT), mTOR: mammalian target of rapamycin, MEK: mitogen-activated protein kinase/ERK kinase, ERK: extracellular-signal-regulated kinase.

The CTLA-4 pathway plays a crucial role in maintaining self-tolerance, and limiting autoimmunity since its downregulation decreases the activation of regulatory T cells (Tregs) and the priming of naive T cells, restricting T cell activation within the secondary lymphoid organs [2,3,4]. CTLA-4 (CD152) is a transmembrane glycoprotein and a CD28 homolog. Its extracellular domain binds to two ligands, B7-1 (CD80) and B7-2 (CD86), whereas its intracellular domain contains a YVKM motif, which is involved in CTLA-4-mediated negative signaling [8]. CTLA-4 is constitutively expressed by Tregs, naive T cells, activated cytotoxic T cells, B cells, natural killers (NKs), and dendritic cells (DCs) [5,7]. CTLA-4 is continuously internalized by endocytosis in Tregs and naive T cells [8]. When antigen-presenting cells (APCs) take up tumor antigens released by apoptotic tumor cells, after breaking them down into peptides, they form their antigen–peptide major histocompatibility complex (MHC). Upon reaching the lymph nodes, the antigen–peptide MHC interacts with the T cell receptor (TCR) of regulatory and naive T cells, as well as the B7-1/2 ligand of APCs with the CD28 stimulatory receptor of T cells. At this point, CTLA-4 gets recycled and translocates to the cell surface due to a negative feedback mechanism caused by CD28 binding. Since CTLA-4 has a higher affinity for B7-1/2, it outperforms CD28 [8,22]. Although there are confounding results regarding the exact implication of signaling pathways due to CTLA-4 activation, it has been established that CTLA-4 recruits Src homology region 2-containing protein tyrosine phosphatase 2 (SHP2). Until now, three potential mechanisms have been proposed for the mode of action of CTLA-4; all three mechanisms involve the RAS/MEK/ERK1/2 pathway and the PI3K/AKT/mTOR pathway, both responsible for T cell survival and proliferation. More specifically, activation of CTLA-4 promotes (1) the direction inhibition of the RAS/MEK/ERK1/2 pathway by SHP2, (2) the inhibition of ZAP70 phosphorylation by SHP2, blocking the PI3K/AKT/mTOR pathway, or (3) the direct inhibition of the PI3K/AKT/mTOR pathway by the YVKM motif [8,22]. Regarding its molecular interactions, several molecules have been identified, including the ring finger protein 19 (RNF19), signal transducer and activator of transcription 5a (STAT5a), tyrosine kinase Lyn (Lyn), Janus kinase 2 (JAK2), receptor tyrosine kinase KIT (KIT), and assembly protein 50 (AP50), among others [8]. CTLA-4 leads to the arrest of T cell activation and proliferation, whereas its blockade promotes T cell activation [2,3,4,5]. T cell activation further induces the production of CD4+ T helper cells, which in turn activate plasma cells and lead to the formation of antibodies, as well as the production of primed CD8+ cytotoxic T cells, which directly attack tumor cells, resulting in cell death, as well as Treg activation within the TME [1,5,6,7,21,23]. Since Tregs are responsible for maintaining self-tolerance, unleashing a crucial inhibitory mechanism may lead to susceptibility to autoimmunity. The signaling mechanisms implicated in CTLA-4 activation are summarized in Figure 1.

**Figure 2:**
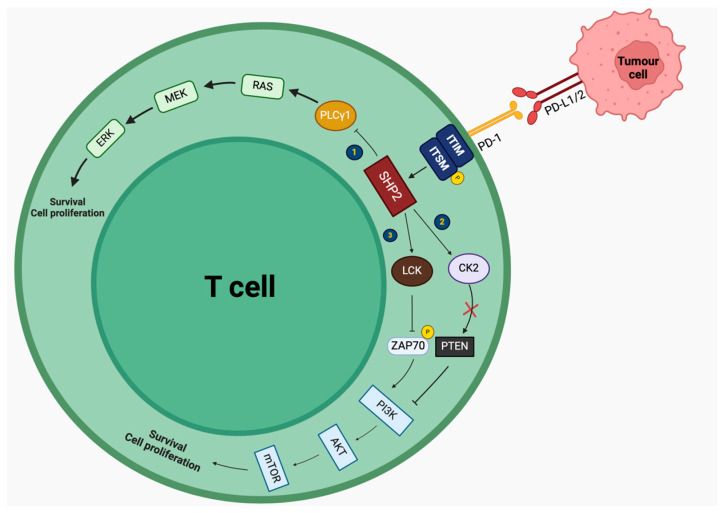
Intracellular mechanisms of PD-1 action in T lymphocytes. Activation of PD-1 decreases T cell proliferation by: (1) inhibiting PLCγ1, leading to the inhibition of the RAS/MEK/ERK1/2 pathway, (2) acting on LCK, inhibiting the phosphorylation of ZAP70 and the activation of the PI3K/AKT/mTOR pathway, and (3) acting on CK2, leading to the phosphorylation of PTEN (presented with an x), inhibiting the PI3K/AKT/mTOR pathway. PD-1: programmed-death 1, PD-L1: programmed death-ligand 1, ITIM: immunoreceptor tyrosine-based inhibitory motif, ITSM: immunoreceptor tyrosine-based switch motif, SHP2: Src homology region 2-containing protein tyrosine phosphatase 2, LCK: lymphocyte-specific protein tyrosine kinase, ZAP70: zeta-chain-associated protein kinase 70, PI3K: phosphoinositide 3-kinase, AKT: protein kinase B (PKB, also known as AKT), mTOR: mammalian target of rapamycin, CK2: casein kinase 2, PTEN: PLCγ1: phospholipase C gamma 1, MEK: mitogen-activated protein kinase/ERK kinase, ERK: extracellular-signal-regulated kinase.

PD-1 is another negative immune regulator expressed on the surface of activated CD4+ and CD8+ T cells, Tregs but also natural killers (NKs), and macrophages [2,5,8]. PD1 (CD279) is a transmembrane protein, whose extracellular domain interacts with two ligands, PD-L1 and PD-L2, expressed by tumor cells, tissue-resident cells, and immune cells, particularly upon interferon-γ (IFN-γ) stimulation [2,3,4]. Its intracellular domain consists of two motifs: immunoreceptor tyrosine-based switch motif (ITSM) and immunoreceptor tyrosine-based inhibitory motif (ITIM). Activation of this pathway results in tyrosine phosphorylation in ITSM, which further recruits SHP2. SHP2 acts on several molecules, disrupting the RAS/MEK/ERK1/2 and PI3K/AKT/mTOR pathways, resulting in the downregulation of effector T cell responses [8,22]. Particularly, SHP2 inhibits the RAS/MEK/ERK1/2 pathway, by (1) inhibiting PLCγ1, and the PI3K/AKT/mTOR pathway by (2) inhibiting the phosphorylation of ZAP70, which is necessary for the activation of the pathway, and (3) by acting on CK2, leading to the phosphorylation of PTEN, a negative regulator of the PI3K/AKT/mTOR pathway, which remains active. On the other hand, PD-1 blockade by ICIs upregulates both effector T cell and NK response, as well as antibody production by B cells against the tumor. Malignant cells expressing PD-L1 take advantage of this interaction, by activating the PD-1 pathway to minimize the immune response against the tumor [1,5,6,7,21,23]. Concerning its molecular interactions, PD-1 has been shown to interact with various molecules, such as Janus kinase 1 (JAK1), integrator complex subunit 7 (INST7), suppressor of cytokine signaling 1 (SOCS1), cell division control protein 45 (CDC45), Tel2 interacting protein 1 (TTI1), protein tyrosine phosphatase non-receptor type 11 (PTPN11), ATP binding cassette subfamily B member 7 (ABCB7), integrator complex subunit 4 (INST4), and anexelekto (AXL), among others [8]. The signaling mechanisms implicated in PD-1 activation are summarized in Figure 2.

Apart from the aforementioned cells and tissues, it is important to note that ICPs are also expressed within the CNS. Specifically, PD-L1 is highly expressed on astrocytes and microglia in the presence of inflammation, whereas PD-1 is constitutively expressed on astrocytes and neurons, and can be potentially expressed on microglia [5,24,25,26,27].

LAG-3 (CD223) is another transmembrane protein, which acts as a negative immune regulator and is similar to CD4 [28,29]. LAG-3 binds with MHC-II with higher affinity than CD4. Its extracellular domain consists of four regions (D1–D4), out of which D1 is the binding site with MHC-II. Apart from MHC-II, other ligands include liver sinusoidal endothelial cell lectin (L-sectin), galactoside-binding soluble lectin (Gal-3), α-synuclein fibrils (α-syn), and fibrinogen-like protein 1 (FGL-1) [8]. Its intracellular domain consists of 3 motifs: KIEELE motif, glutamate-proline dipeptide multiple repeats motif (EP), and serine phosphorylation motif (S484) [8,28,29]. LAG-3 cross-links with the TCR/CD3 complex and it also blocks calcium influx [8]. However, the exact mechanisms involved in T cell inhibition remain elusive, until now. LAG-3 interacts with the F-box only protein (FBXO), S-phase kinase associated protein 1 (SKP1), protocadherin-20 (PCDH20), neuronal nicotinic acetylcholine receptor subunit alpha-5 (CHRNA5), and protocadherin-7 (PCDH7), among others [8].

## 4. Pathogenesis of Neurological irAEs Caused by ICIs

ICIs, by acting on specific ICPs, aggravate immune responses either against a tumor-associated antigen or a tumor-specific mutation, resulting in exacerbated T cell activation and antibody production [6,17]. Several mechanisms have been proposed to be involved in the development of neurological irAEs caused by ICIs. Both direct and indirect mechanisms of neurotoxicity may be implicated, including systemic, local, and autoimmune processes. Furthermore, the pathogenesis of various neurological irAEs may rely on the synergistic effect of several underlying mechanisms of neurotoxicity [6,21].

Circulating CD8+ cytotoxic T cells and antibodies cause neurotoxicity via cell-dependent or complement-dependent cytotoxicity since they can easily affect the nervous tissue outside the CNS [21]. Therefore, unprotected tissue outside the blood–brain barrier (BBB) is exposed to neurotoxicity, caused by this aggravated immune response. This mechanism is a possible explanation for the higher incidence of irAEs affecting the periphery compared with those affecting the CNS. However, neurotoxicity of the CNS can be facilitated by any causative factor resulting in BBB disruption, allowing for the influx of various molecules, particularly in the presence of local CNS inflammation, for instance, due to brain metastatic disease. Consequently, all individuals with brain metastases receiving ICI regiments are susceptible to direct CNS neurotoxicity and, therefore, the development of irAEs [6].

Local CNS inflammation with subsequent BBB disruption could also explain the progression of radiologically isolated syndrome (RIS) to multiple sclerosis (MS), as well as the exacerbation of pre-existing MS [21,30]. Any disruption of the BBB could allow ICIs to enter the CNS and bind to their target molecules (PD-1 and PD-L1), expressed on CNS resident cells, due to local inflammation, aggravating a pre-existing condition. Additionally, it has been suggested that the influx of ICI-altered lymphocytes from the periphery into the CNS may exacerbate local CNS injury. In experimental autoimmune encephalomyelitis (EAE), the experimental model of MS, EAE mice presented with increased disease severity and progression following ICI administration. According to the results, PD-1 expression was significantly higher in CD4+ T cells with the highest affinity with myelin oligodendrocyte glycoprotein (MOG) [31]. Therefore, clinical progression could be attributed to the uncontrollable influx of high-affinity T lymphocytes.

Regarding the occurrence of paraneoplastic neurological syndromes following ICI administration, similarly to the development of spontaneous paraneoplastic neurological syndromes, tumor-associated antigens or tumor-specific mutations against which antibodies are formed (aberrant expression), might also be physiologically expressed in the nervous system (physiological expression) [5,6,21]. Autoantibodies targeting extracellular proteins have been proven to be pathogenic; specifically, they bind to synaptic receptors, located on the cell surface, and they either functionally block them, by preventing other molecules from binding, or internalize them, altering the synaptic density of the cell. However, autoantibodies targeting extracellular proteins do not seem to cause cell death, and the irAEs caused by these antibodies can be reversed, if the responsible autoantibodies become depleted [24]. On the other hand, the mechanism of pathogenicity of autoantibodies targeting intracellular antigens remains unknown. However, the presence of intracellular antibodies is associated with a worse prognosis [24]. It is important to note that in the vast majority of cases, paraneoplastic antineuronal antibodies are present in the serum and rarely in the cerebrospinal fluid (CSF), whereas the BBB is considered to be intact [32].

In a mouse model of paraneoplastic cerebellar degeneration, scientists inserted a breast tumor bearing a neo-self antigen that was concomitantly expressed on Purkinje neurons. Consecutively, they administered primed lymphocytes against the neo-self antigen to one group of mice and a CTLA-4 inhibitor to the other group. Only those mice treated with the ICI exhibited neurological manifestations, whereas sole primed lymphocyte administration was unable to trigger the appearance of neurological symptoms. Moreover, histopathological analysis revealed marked antigen-specific CD8+ T cell cerebellar infiltration, whereas autoantibodies did not play a significant role. According to the results, Purkinje neuronal cell loss was associated with CD8+ T cells [33]. Although this model does not explain the pathophysiological mechanism of spontaneous paraneoplastic syndromes, it provides valuable insight regarding post-ICI paraneoplastic neurological syndromes and their association with the exacerbation of systemic immune responses. Consequently, although antigen aberrant expression is necessary for the development of paraneoplastic syndromes, it is not the trigger of neurological manifestations.

Another clue supporting that sole aberrant expression is incapable of developing paraneoplastic neurological syndromes is that SCLC constitutively expresses Hu antigens. However, anti-Hu antibodies are present only in a limited number of individuals (16%), whereas only 1–3% develop paraneoplastic neurological syndromes, similar to the incidence of anti-Yo related paraneoplastic neurological syndromes in women with gynecological tumors [34,35,36]. Mutations leading to anti-Yo overexpression were found in all tumors of women presenting with paraneoplastic neurological syndromes and detectable anti-Yo antibodies, whereas these mutations were absent in tumors of negative individuals [37]. Therefore, antigen overexpression by the tumor may lead to the disruption of self-tolerance and the development of paraneoplastic neurological syndromes. Finally, the allele HLA-B*27:05, which is rarely found in the general population, was identified in three out of five individuals with meningoencephalitis, treated with ICIs. This allele has been previously associated with other immune-mediated disorders, including ankylosing spondylitis and psoriasis, and could be a potential risk factor for the development of paraneoplastic neurological syndromes [38,39].

Yet, today, the exact underlying pathophysiological mechanisms involved in the development of neurological irAEs remain elusive. However, the broad administration of ICIs has significantly increased the incidence of rare neurological diseases. The increased incidence of rare disorders, as well as the exacerbation of immune responses, may provide valuable information regarding the pathogenesis of these diseases.

## 5. Clinical Presentation of Post-ICI Neurotoxicity

Neurological irAEs of all grades are estimated to occur in ~1% of individuals under monotherapy and up to 5% of individuals receiving combination treatment [16,40]. Neuromuscular disorders are encountered three times more frequently and appear with a shorter latency compared to CNS adverse events. The majority of post-ICI neurological irAEs have distinctive features, which distinguish them from their idiopathic counterparts [40]. Isolated and non-specific neurological symptoms, such as fatigue, puzzlement or confusion, distress, headache, and discomfort are excluded from the recently established classification of ICI adverse events [41].

### 5.1. Neurological Disorders of the PNS

#### 5.1.1. Myositis

Post-ICI myositis can either emerge de novo or as a reactivation of a pre-existing autoimmune disorder, such as polymyositis or dermatomyositis [42]. It is the most frequent neurological irAE, accounting for 32% of cases [40,43]. Typically, the onset occurs within 2 months following ICI therapy initiation, and it has been mostly associated with anti-PD-L1 agents [17,40].

Myositis presents with a fixed pattern of muscular weakness [41]. Proximal limb muscles are more affected than the distal ones, leading to limitations in ambulation, raising objects, and lifting arms, whereas axial weakness, particularly in the cervical region, results in difficulties in neck extension and flexion (drop head syndrome). Oculobulbar involvement, characterized by blepharoptosis, diplopia, dysphagia, dysarthria, and respiratory difficulties is a distinct and prominent feature of post-ICI myositis, being the primary or only manifestation in ~40–50% of patients, in contrast with the idiopathic counterparts [40,44]. Ocular symptoms may fluctuate, mimicking post-ICI myasthenia gravis (pseudo-myasthenia) [45]. Muscular pain generally occurs only in severe myositis cases and sometimes precedes muscular weakness and creatinine kinase (CK) elevation. Dermatomyositis associated with cutaneous symptoms, such as heliotrope rash and Gottron papules, rarely occurs with ICI therapy [46].

CK levels are usually elevated, but do not correlate with the severity of muscular weakness; however, they remain normal in up to 30% of cases [44]. In individuals with normal CK levels, aldolase concentrations should be determined, as high levels have been reported in the absence of CK elevation, providing additional diagnostic insight [40]. Electromyography (EMG) usually detects myopathic motor unit potentials with positive sharp waves and fibrillation potentials, but normal findings have also been reported in up to 20% of cases [40,43,44]. Decremental patterns, indicative of neuromuscular junction dysfunction, are absent in individuals with myositis [40]. Myasthenia-specific antibodies (acetylcholine receptor (AChR), muscle-specific kinase (MuSK), low-density lipoprotein receptor (LRP4)) may be positive, indicating the presence of concurrent myasthenia, especially when ocular or bulbar symptoms are predominant [44,47]. Anti-striational antibodies are positive in nearly half of patients (~55%) [45,47]. On the other hand, myositis-specific antibodies, such as anti-nuclear antibodies (ANA), Jo-1, PL-7, PL-12, and EJ, are usually absent [44]. Limb magnetic resonance imaging (MRI) with the use of a contrast medium typically reveals short tau inversion recovery (STIR) hyperintensities or contrast enhancement in the affected muscles [41]. Muscle biopsy is only recommended in cases of doubtful diagnosis and reveals a specific pattern of inflammatory necrotic myositis with focal clusters of necrotic tissue and immune-cell infiltrates (mainly T lymphocytes and macrophages), scattered across the endomysium of the affected muscles [44]. Up to 75–80% of individuals improve after ICI discontinuation and subsequent corticosteroid treatment initiation [44]. However, half of them (~40%) experience recurrent relapses, within the following years [43,46,48].

The overlap of myositis, myocarditis, and myasthenia gravis in ICI-treated individuals has been reported in approximately 50% of myositis cases [49]. This triad is associated with poor prognosis, with a mortality rate reaching 60% [50]. Therefore, routine screening for myocarditis, with troponin levels and repeated electrocardiographs (ECGs), and myasthenic symptoms is mandatory in all individuals presenting with post-ICI myositis.

#### 5.1.2. Myasthenia Gravis (MG)

Post-ICI MG occurs in about 14% of cases and tends to develop exceptionally early, usually within the first month, whereas in some cases, as early as 1 week after ICI initiation [51,52]. It is more likely to develop in individuals receiving anti-PD-L1 therapy [51]. MG should be suspected in individuals exhibiting fluctuating, activity-dependent muscle weakness, predominantly involving the proximal and axial cervical muscles, ocular muscles (diplopia, external ophthalmoplegia, asymmetrical blepharoptosis), and bulbar muscles (dysphagia, dysarthria, dyspnea). Respiratory muscle weakness is far more frequent in post-ICI MG compared with the spontaneous forms, accounting for over 50% of cases [43,52].

The ice pack test aids the diagnosis, by reducing cholinesterase activity. Nerve conduction studies show a decremental pattern and reduction in action potentials in repeated nerve stimulation, which is a hallmark of MG (both idiopathic and post-ICI) [53]. AchR autoantibodies are detected in approximately 70% of cases [43,54]. If negative, Musk and LRP4 antibody testing should be considered [43].

Although most patients improve, fatality can reach up to 30%, primarily due to respiratory muscle failure and concomitant acute myocarditis [43]. Long-term immunosuppression is often necessary, due to frequent relapses, apart from individuals presenting with minor manifestations, confined to the ocular muscles [55]. Distinguishing between MG and myositis can be challenging, especially in cases of myositis with predominant oculobulbar symptoms [40]. Clinical fluctuations and EMG decremental patterns are suggestive of MG, while CK elevation and pain are signs of myositis.

#### 5.1.3. Guillan–Barré Syndrome (GBS) and Other Neuropathies

Post-ICI neuropathies appear in 22% of individuals and encompass a broad spectrum of disorders, including acute demyelinating polyradiculoneuropathies, such as GBS and rare variants (acute inflammatory demyelinating polyneuropathy (AIDP), Miller Fisher syndrome (MFS), acute motor axonal neuropathy (AMAN), acute motor and sensory axonal neuropathy (AMSAN), chronic inflammatory demyelinating polyneuropathy (CIDP)) [40,44,56]. Less common manifestations include plexopathies, small-fiber neuropathies, multiple mononeuropathies, carpal tunnel syndrome, neuralgic amyotrophy, and cranial neuropathies, affecting the optic, trigeminal, abducens, facial, vestibulocochlear, and glossopharyngeal nerves, often bilaterally [40,43,57,58,59,60].

The incidence of post-ICI GBS is less than 1%, typically occurring within 6 months of treatment initiation, although cases of delayed onset have also been reported [47]. The diagnosis of GBS and its variants is primarily clinical. Key patterns include rapidly progressive, (mostly) symmetrical, ascending muscle weakness with areflexia. Weakness varies from mild walking difficulties to complete paraparesis. Sensory symptoms (paresthesia, numbness, neuropathic pain in the lower limbs or lumbar region) and autonomic dysfunction (blood pressure fluctuations, urinary and bowel retention) are usually present. Cranial nerve involvement, bulbar symptoms, and dyspnea are atypical characteristics [43].

Nerve conduction studies usually reveal a demyelinating pattern, indicative of acquired polyradiculoneuropathy, with decreased motor unit velocities, conduction block, and prolonged F-wave latencies. Swelling of the respective nerve roots impairs CSF flow, leading to albuminocytologic disassociation. Non-specific MRI hyperintensities or gadolinium enhancement of cauda equina roots, and/or cranial nerves have also been described. Anti-ganglioside and neuronal antibodies are typically absent in ICI-induced GBS [61].

Post-ICI GBS is more likely to present acutely, without a temporal relationship with an infection, and has an excellent response to corticosteroid treatment [42,43,55]. Mortality rate reached up to 10%, due to respiratory failure, caused by cervical nerve root involvement [35].

### 5.2. Neurological Disorders of the CNS

#### 5.2.1. Encephalitis

The most lethal manifestation is encephalitis, accounting for almost 13% of all neurological irAEs [40,43]. However, this term cumulates a relatively heterogenous spectrum of disorders, with distinct clinical presentations, antibody and tumor associations, and therapeutic outcomes [40]. Associations with lung cancer and PD-L1 treatment have been reported [43].

#### 5.2.2. Meningoencephalitis

This term describes a rather diffuse CNS dysfunction [40]. Most patients develop an acute/subacute decrease in mental status, isolated or accompanied by lethargy, decreased alertness, fever, meningeal signs (photophobia, nausea, vomiting, cervical stiffness), seizures, or less often, aphasia, tremor and opsoclonus-myoclonus [21,62,63]. CSF testing shows pleocytosis, increased albumin content, and oligoclonal bands, with higher cell count and protein levels than focal syndromes [64]. Brain MRI is unrevealing in up to 50% of cases; however, leptomeningeal contrast enhancement and brain parenchymal T2 hyperintensities have been seen in a subgroup of cases [65,66]. Paraneoplastic antineuronal antibodies are usually absent; however, glial fibrillary acidic protein (GFAP) antibodies have been detected in a few cases [62,67]. Despite disease severity at the peak of clinical symptoms, the prognosis is usually favorable following ICI withdrawal and immunosuppressive treatment initiation [66]. So far, the safety of ICI reintroduction remains unclear. According to a few currently available observational studies, relapse rates reach up to 30% [67].

#### 5.2.3. Limbic Encephalitis

Individuals with limbic encephalitis present with the classical clinical triad of symptoms: subacute anterograde amnesia, temporal lobe complex partial seizures, and psychiatric symptoms (behavioral/personality alterations) [64,66,68,69]. Paraneoplastic antineuronal antibodies, particularly anti-Hu and anti-Ma2, are frequently detected. Of note, anti-Ma1 and anti-Ma2 encephalitis may also be accompanied by diencephalic symptomology, resembling narcolepsy-cataplexy syndrome [70]. CSF analysis may reveal inflammation and pleocytosis, although this finding is less frequently encountered in limbic encephalitis compared with diffuse meningoencephalitis. MRI usually demonstrates contrast enhancement in the amygdaloid nucleus and hippocampus, often bilaterally [66].

Usually, individuals with limbic encephalitis, particularly those bearing high-risk paraneoplastic antibodies, such as anti-Hu, respond poorly both to corticosteroid treatment and second-line immunosuppressants, such as cyclophosphamide [66]. The mortality rate is strikingly high, reaching up to 75%. The combination of several factors, including the severity of neurological symptoms, the presence of an aggressive underlying malignancy, the advanced stage of the tumor, and the removal of the ICI in the shadow of severe neurotoxicity contribute to this high number [17].

#### 5.2.4. Rapidly Progressive Cerebellar Encephalitis

Post-ICI cerebellar ataxia is a rare irAE, representing only 1% of all neurotoxicities [43]. Individuals present with an acute/subacute onset of rapidly progressing cerebellar symptoms, including ataxic dysarthria, gait imbalance, limb/truncal ataxia, dysmetria, vertigo, and nystagmus [64,66,71]. Cerebellar ataxia may occur either in isolation (1/3 of cases) or in the context of more complex clinical entities, including limbic and brainstem symptoms, such as cranial neuropathies and long track involvement [64]. Antineuronal autoantibodies typically associated with post-ICI cerebellar ataxia include anti-Hu and anti-PCA2, among others, whereas antibodies predominantly associated with the idiopathic or spontaneous paraneoplastic cerebellar ataxia, such as anti-Yo, anti-Ri, and anti-DNER (Delta and Notch-like epidermal growth factor-related) are rarely detected in the post-ICI context [71,72].

In most cases, CSF analysis shows inflammation, pleocytosis, elevated protein, and oligoclonal bands. MRI scans may be helpful, revealing cerebellar abnormalities (in 40% of individuals) and sometimes cerebellar atrophy [71]. In general, the detection of antineural autoantibodies carries a poor prognosis [35,73]. However, in a recently published systematic review, high rates of recovery and clinical improvement, up to 67%, have been reported [27].

#### 5.2.5. Aseptic Meningitis

Meningeal inflammation represents only 3% of all ICI-neurotoxicities; however, mild, oligosymptomatic, or atypical cases may remain undiagnosed [43]. Aseptic meningitis has been associated with younger age (<50 years old), female sex, melanoma, and CTLA-4 inhibitors. Individuals typically present with new-onset or unusual and persistent headaches, often with photo-/phonophobia and fever [43,51]. In the presence of concurrent mental status decline, indicating brain parenchyma involvement, the term meningoencephalitis is preferred. CSF abnormalities usually include minor to moderate lymphocytic pleocytosis (>90% of cases), elevated albumin levels, and intrathecal oligoclonal bands. Brain MRI is usually normal; however, meningeal enhancement and T2 hyperintensities may be present in 30% of cases [16,43]. Antibody testing for the detection of antineuronal antibodies is usually negative. The disease prognosis is excellent; the majority of affected individuals recover completely after steroid treatment, while spontaneous recovery has also been reported after ICI discontinuation [43].

#### 5.2.6. Longitudinal Extensive Transverse Myelitis (LETMS)

Post-ICI myelitis is quite uncommon, accounting for 2% of all ICI-induced neurotoxicities [43]. It has been mostly associated with PD-L1 inhibitors and combined therapies. Nearly all affected individuals develop motor weakness in the lower limbs, tactile/thermic sensory disturbances, paresthesias, proprioceptive ataxia, and sphincter dysfunction. In most cases, spinal MRI reveals extensive longitudinal lesions (>3 segments in length) and variable contrast enhancement on brain and spinal MRI scans (diffuse hyperintensities on the spinal cord, brain parenchyma, meninges, and cauda equina roots) [74,75]. In CSF analysis, pleocytosis, elevated protein levels, and oligoclonal bands are usually present in up to 50% of cases; this finding may be helpful in the differential diagnosis between ICI-mediated myelitis and chemotherapy-induced myelopathy [75]. Anti-GFAP antibodies may be detected, whereas anti-MOG or aquaporin-4 (anti-AQP4) antibodies are usually negative [43,74,76]. The vast majority of individuals (~70%) have a favorable response to corticosteroids. However, early severe relapses during oral prednisone tapering are frequently reported, and, therefore, second-line agents are commonly needed (cyclophosphamide, tocilizumab, etc.) [43,75].

#### 5.2.7. Other Demyelinating Disorders

The incidence of post-ICI demyelinating disorders is less than 0.5% [51]. ICI therapy has been associated with both de novo demyelination, such as the appearance of radiologically RIS, MS, or acute disseminated encephalomyelitis (ADEM), and exacerbations of pre-existing conditions, including MS and neuromyelitis optica spectrum disorder (NMOSD) [77,78,79,80,81,82]. Symptoms vary according to the lesion site. Brain MRI typically demonstrates enhancements and hyperintense T2/FLAIR lesions. Lumbar puncture usually reveals lymphocytic pleocytosis, elevated protein levels, and oligoclonal bands. While most individuals test negative for these, AQP4, MOG, collapsing response-mediator protein-5 (CRMP5), and Hu, among others, have been detected in a few cases [75]. Most individuals recover, at least partially, after ICI interruption [80].

### 5.3. Autoimmune Movement Disorders

Post-ICI movement disorders are rare (3% of cases), and they always have an acute onset, which represents a major diagnostic clue, raising clinical suspicion for ICI neurotoxicity [71]. Furthermore, they scarcely occur in isolation but rather in the context of ICI-mediated encephalitis, meningitis, or peripheral neuropathy. Clinical phenotypes include tremor, parkinsonism, myoclonus, chorea, hemiballismus, akathisia, and stiff-person syndrome [83]. Brain MRI demonstrates non-specific basal ganglia T2/FLAIR abnormalities, but it may also be normal. CSF analysis, apart from inflammation, in about 50% of cases reveals the presence of antineuronal autoantibodies. Antibody-negative individuals have an excellent response to corticosteroids [83].

## 6. Diagnostic Approach to Neurological Investigation

The majority of neurological irAEs present as new-onset, acute neurological symptoms, in close temporal relationship with the initiation of ICIs [40,43]. Most irAEs occur within the first 2–6 months of treatment [40,64,84]. Although late-onset neurological side effects have been scarcely reported, the occurrence of neurological symptoms beyond 6–12 months from the last ICI infusion should raise suspicion for an alternative diagnosis [40,41].

The first step in the diagnostic approach is to exclude alternative causes of neurological dysfunction, including direct and indirect effects of cancer (metastatic disease of the CNS, carcinomatous leptomeningeal disease, and/or coagulopathy, metabolic imbalance, etc.), toxicity of concurrent cancer treatments (susceptibility to neurotoxic infectious agents, or posterior reversible encephalopathy syndrome (PRES)), and longitudinal side effects of previous radiotherapy/chemotherapy (cisplatin-induced sensory neuropathy, radiation-induced persistent migraine attacks) [40].

The next step to support the diagnosis of post-ICI neurotoxicity is to provide laboratory or radiological evidence of ongoing neuroinflammation [40,43]. Paraneoplastic antineuronal antibody testing is recommended. However, the results should always be interpreted in the context of the clinical presentation, as patients with certain cancer types, such as SCLC, may asymptomatically harbor these antibodies (e.g., anti-Hu) [85].

**Figure 3:**
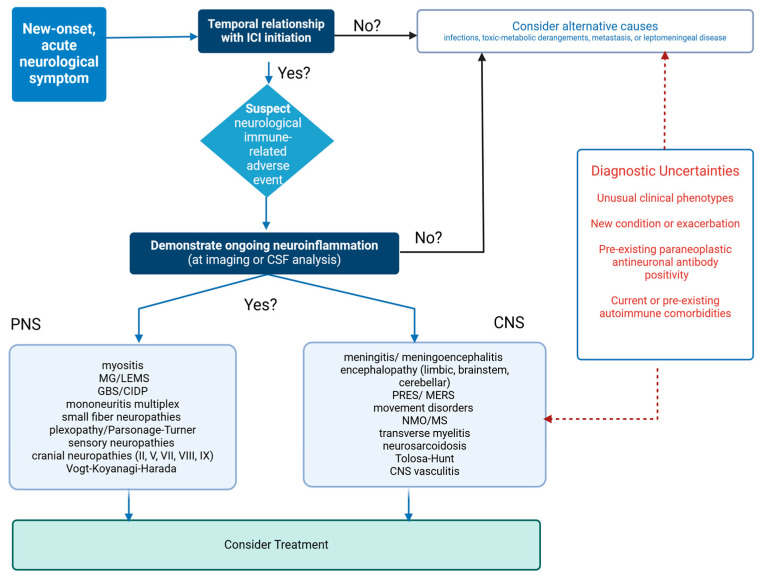
Diagnostic algorithm for individuals receiving ICIs, presenting with new-onset neurological symptoms. ICI: immune checkpoint inhibitor, CSF: cerebrospinal fluid, MG/LEMS: myasthenia gravis/Lambert Eaton myasthenic syndrome, GBS/CIDP: Guillain-Barré syndrome/chronic inflammatory demyelinating polyneuropathy, PRES/MERS: posterior reversible encephalopathy syndrome/mild encephalopathy with reversible splenial lesion, NMO/MS: neuromyelitis optica/multiple sclerosis, CNS: central nervous system.

Concurrent non-neurological irAEs and oncological response to treatment may both increase the likelihood of a neurological irAE diagnosis [57]. Once the suspicion of neurotoxicity is established, an effort should be made to attribute the symptoms to one of the well-defined syndromes analyzed above, as they are related to different treatment and mortality outcomes (Figure 3).

## 7. Treatment Options

The management of neurological irAEs is poorly established. Current guidelines are based on retrospective studies and expert opinions alone, emphasizing the need to view the proposed agents and dosing as suggestive rather than conclusive [47]. According to international oncology guidelines, Grade 1 neurological irAEs (which include mild symptoms that do not interfere with daily activities) may permit the continuation of ICI treatment in selected cases. However, caution is needed due to potential rapid escalation to Grade 2 severity level [42,55].

As with non-neurological irAEs, the initial treatment step often involves ICI withdrawal and subsequent corticosteroid therapy initiation [42,55]. Corticosteroids form the cornerstone of post-ICI neurotoxicity management, and they are effective even for conditions in which steroids are not usually recommended, such as GBS and parkinsonism [16,42,55]. An initial dosage regimen is oral prednisone (1 mg/kg) for Grade 2 symptoms and high-dose intravenous methylprednisolone (1 g daily for 3–5 days) for severe neurotoxicity (Grades 3 and 4). In case of improvement, transition to oral steroids with a prolonged period of tapering for 3–12 weeks is recommended, with consideration of steroid-related side effects on tumor evolution. Short courses of corticosteroids do not hamper tumor control, but the safety of long-term treatment with corticosteroids or other immunosuppressants is yet to be determined [57].

Unfavorable treatment outcomes are reported in about 30% of cases, and the risk factors include advanced age, aggressive malignancy, high severity of symptoms at onset, concurrent myocarditis, CNS involvement (particularly, focal encephalitis), presence of antineuronal antibodies targeting intracellular antigens, and ICI administration in individuals with previous paraneoplastic autoantibodies and/or autoimmune disorders [66,73,86,87,88,89]. In case of corticosteroid resistance (lack of response within 10–14 days of treatment), and in individuals with negative prognostic factors, as well as in all cases of GBS and MG, rapid escalation to intravenous immunoglobulins (IVIG) (2 mg/kg/day for 3–5 days) or plasmapheresis (5–7 sessions) is advocated [47].

The choice between IVIG and plasmapheresis should be personalized. IVIG is more readily available in hospital settings and requires less monitoring compared to plasmapheresis, which should be preferred in serious, acute cases due to the faster onset of action. Given the long half-lives of ICIs (3–4 weeks), leading to a prolonged effect long after their withdrawal, plasma exchange can accelerate their clearance [90]. Contraindications for each option should also be considered (Table 1).

**Figure 4:**
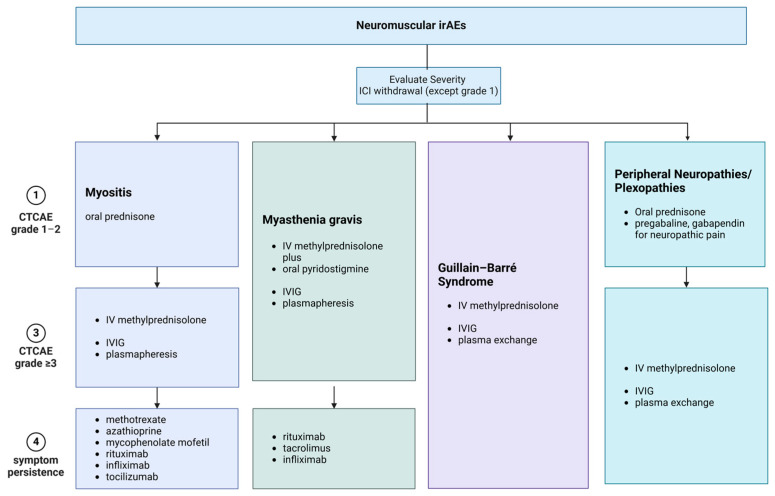
Treatment options for the most frequently encountered ICI-irAEs, affecting PNS. Treatment options vary among different disorders, and according to disease severity and response to treatment. All currently available treatment options for neurological irAEs affecting the PNS are summarized in this figure. irAEs: immune-related adverse events, ICI: immune checkpoint inhibitor, iv: intravenous, IVIG: intravenous immunoglobulin.

**Figure 5:**
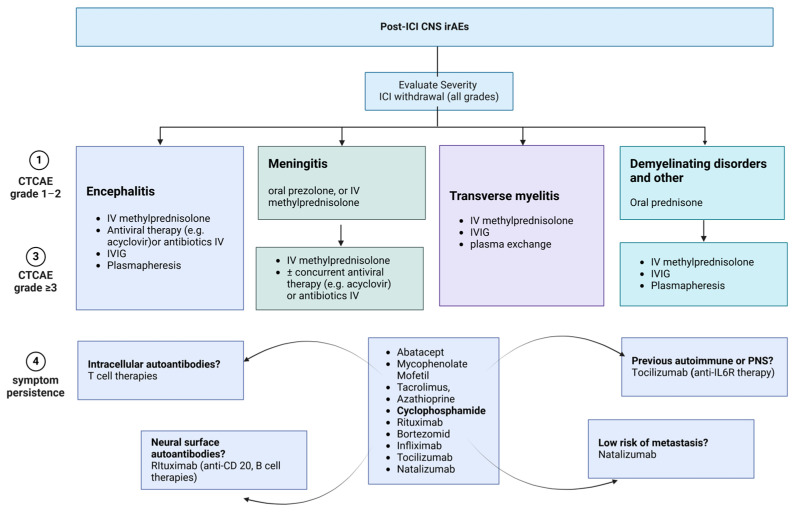
Treatment options for the neurological irAEs, caused by ICI administration, affecting the CNS. Treatment options vary among different disorders, and according to disease severity, personal history, response to treatment, and the presence of autoantibodies. Therefore, the choice of appropriate treatment depends on several factors and should be personalized for each individual. All currently available therapies for CNS irAEs are summarized in this figure. ICI: immune checkpoint inhibitor, CNS: central nervous system, irAEs: immune-related adverse events, iv: intravenous, IVIG: intravenous immunoglobulin, anti-IL6R: interleukin-6 receptor antibody, anti-CD20: cluster of differentiation 20 antibody.

In refractory or relapsing cases, escalation to second-line therapies is prudent and includes options such as abatacept, mycophenolate mofetil, tacrolimus, azathioprine, cyclophosphamide, rituximab, bortezomib, infliximab, tocilizumab, and natalizumab. The optimal choice should be guided by the presumed pathophysiological mechanism of the disorder and an individual’s comorbidities [87]. Figure 4 and Figure 5 summarize the recommended management of common neurological irAEs.

Overall, the management of corticosteroid refractory cases is poorly defined and should weigh up the expected benefits in residual disability against the risk of cancer evolution posed by each therapeutic agent. More research on the underlying immune-mediated pathophysiology is needed to design optimal treatment strategies for each type of neurological irAEs.

## 8. ICI Rechallenge

Resuming ICI after neurological recovery is a matter of debate [91]. ICI re-initiation may lead to a recurrence of neurological symptoms [92]. However, most individuals have advanced-stage malignancies, with limited alternative therapies available, and, therefore, treatment with ICIs may be crucial for the oncological management and survival of the patient [66,89,93].

The decision on whether or not ICI therapy should be re-administered depends on the severity of the neurological disorder that subsided, the extent of recovery, and the status of the malignant disease [65]. It may be reasonable to consider re-initiating ICI therapy if neurological side effects do not exceed Grade 1 (or even 2; although, with caution), and the symptoms resolved completely with corticosteroid therapy since relapse rates in mild cases are reported relatively low [94]. For severe irAEs (grade ≥ 3), such as GBS, MG, concurrent myocarditis, focal encephalitis or transverse myelitis, and partial recovery with second-line prolonged immunosuppressive therapies, clinicians should consider permanent ICI withdrawal [14]. Overall, the decision to rechallenge must be taken on a case-by-case basis, ideally by a multidisciplinary team, also involving the patients and the caregivers.

## 9. Discussion

Nowadays, ICIs are being used for the treatment of more than 20 advanced and aggressive malignancies with exceptional results, increasing both survival and long-term remission rates. However, these outstanding results are obtained at the expense of a wide spectrum of irAEs. The blockade of critical inhibitory pathways of the immune system leads to a widespread immune system activation with cytotoxic T cell and antibody formation [1,6,40,62]. Since these events occur in the periphery, neurotoxicity occurring outside the CNS is easily comprehended and explains the higher incidence of neurological irAEs affecting the neuromuscular junction compared with those affecting the CNS. Furthermore, local CNS inflammation, leading to the disruption of the BBB, may allow for the influx of immune cells and ICIs in the CNS. BBB disruption will give the opportunity to abundant ICI-altered T lymphocytes and antibodies to enter the CNS, whereas ICIs may inhibit the pathways of their target molecules, PD-1 and PD-L1, potentially expressed in CNS tissue-resident cells, due to inflammation, aggravating the pre-existing CNS damage [31].

However, the pathogenic mechanism of post-ICI paraneoplastic neurological syndromes raises several important questions, which need to be addressed. Prior to the development of ICIs, the pathogenesis of paraneoplastic neurological syndromes was largely attributed to aberrant antigen expression by certain types of malignancies. However, the introduction of ICIs doubled the incidence of these syndromes and revealed the unusual occurrence of paraneoplastic neurological syndromes in individuals with tumors previously unrelated to these manifestations, such as melanoma and renal cell carcinoma [6,21]. Potentially, increased epitope spreading due to enhanced drug-induced T lymphocyte cytotoxicity against the tumor may reveal paraneoplastic antigens, which would have otherwise remained hidden or slightly expressed. Moreover, although SCLC constitutively expresses Hu antigens, one would expect that a significant portion of individuals with SCLC receiving ICIs would present with anti-Hu antibodies and exhibit neurological symptoms. However, only an unexpectedly low number of individuals present with neurological manifestations, indicating that aberrant expression alone is unable to lead to the development of these disorders. In addition to that, the presence of asymptomatic antibody-positive individuals also highlights this gap [35]. Similarly, in the animal model of paraneoplastic cerebellar degeneration, aberrant expression in the absence of an aggravated immune response caused by ICI administration did not trigger the appearance of neurological symptomatology [33]. Although these results are inconsistent with the development of spontaneous paraneoplastic neurological symptoms, they demonstrate that CNS injury is primarily caused by exacerbated T cell cytotoxicity, enriching our knowledge regarding disease pathogenesis and explaining the involvement of CNS despite the intact BBB. The presence of mutations leading to overexpression of Yo antigens in gynecological tumors of female individuals presenting with paraneoplastic neurological syndromes, which were absent in individuals without neurological symptoms, could be a potential risk factor in both spontaneous and post-ICI cases; antigen overexpression may overcome self-tolerance [36,37]. Additionally, the identification of a rarely encountered allele in three out of five individuals with post-ICI meningoencephalitis indicates that genetic predisposition may also be implicated [38,39]. Although ICIs are administered in advanced stages and aggressive types of malignancies, the possibility of developing a potentially fatal neurological disorder should be taken into consideration prior to ICI administration. Currently, there are no available prospective studies on antibody-positive individuals prior to ICI administration, receiving immunotherapy. Probably, a certain number of individuals would benefit from this testing and oncologists should consider antibody testing in individuals with tumors strongly associated with paraneoplastic neurological disorders, although the cost–benefit of this process should also be established. Despite the differences between spontaneous and post-ICI paraneoplastic neurological syndromes, research may benefit from the increased incidence of these rare disorders by gathering new findings, which might further explain the pathogenesis of both spontaneous and post-ICI disease, unraveling currently unidentified antibodies, establishing novel, more accurate methods of antibody detection and developing more efficient, targeted treatments.

Most neurological irAEs comprise clinically distinct syndromes, with different responses to treatment and prognosis, and most probably, different underlying pathogenic mechanisms. Currently, myositis, sometimes with concurrent myasthenic symptoms and myocarditis, and neuropathies, including GBS and variants, are the most common clinical presentations. Although CNS involvement is less frequently seen, it carries a significant risk of long-lasting disability and mortality [43,52,87,95].

Diagnosis of post-ICI neurotoxicity relies on the exclusion of alternative diagnosis and the close temporal relationship (2–6 months) between symptoms and ICI initiation. According to universal guidelines (level of evidence V), suspension of the inhibitor and administration of corticosteroids are essential first steps in the management of neurological irAEs, while the administration of IVIG or plasmapheresis is robustly implicated in MG, GBS, and encephalitis when more aggressive interventions are warranted. The management of refractory cases, however, is yet to be established. Several immunosuppressant and immunomodulatory agents have been used in various retrospective studies and case reports, but the optimal choice for each clinical phenotype remains elusive, especially for patients with pre-existing autoimmune diseases or paraneoplastic antibodies [40,55,94]. Rituximab, for example, has already been used successfully in the context of N-methyl-D-aspartate receptor (NMDAR) or contactin-associated protein-like 2 (CASPR2) encephalitis, which supports the future application of more recent humanized anti-CD20 antibodies, such as ofatumumab or ocrelizumab, in post-ICI neurotoxicity. Future studies will clarify optimal treatment strategies for individuals with ICI-related neurological autoimmunity, as well as the safety of ICI re-initiation in individuals achieving neurological recovery.

The high prevalence of irAEs highlights the need for the identification of reliable biomarkers, which could both predict the development of irAEs and facilitate the follow-up of these diseases. Serum proteins, complete blood count (CBC), cytokines, antibodies, HLA genotypes, microRNA, and gene expression profiling have been examined [96,97]. Until now, only serum proteins and CBC have revealed some useful results. A sudden increase in C-reactive protein (CRP), as well as a decrease of white blood cells (WBCs) and lymphocytes compared with baseline could potentially predict the occurrence of an irAE. Additionally, increased thyroid stimulating hormone (TSH) and troponins, as well as the presence of rheumatoid factor at baseline have been associated with the development of irAEs [97]. Regarding neurological irAEs, autoantibodies are being examined as potential disease biomarkers [96,98]. Although autoantibodies targeting the neuromuscular junction have increased sensitivity and specificity in the diagnosis and, therefore, they could potentially predict the occurrence of an irAE, no clear association has been found between brain-reactive autoantibodies and the occurrence of neurological irAEs, until now [98].

## 10. Conclusions

Although the incidence of severe neurological irAEs caused by ICI administration is low, they carry a high disability burden and, in some cases, they can also be fatal. Therefore, prompt action needs to be taken to ensure an accurate diagnosis and proceed to proper management. Yet, today, important questions regarding the pathogenesis of these irAEs remain unanswered. Further research is needed for the identification of currently unidentified antineuronal autoantibodies, as well as reliable and widely accessible biomarkers. Large-scale prospective studies examining the role of antineuronal antibodies in antibody-positive individuals receiving ICIs are required. Finally, the establishment of more accurate qualitative and quantitative methods of antibody testing is necessary and would be beneficial for the development of targeted treatment. The increased incidence of rare neurological disorders due to ICI administration gives neuroscientists a chance to explore the complex mechanisms implicated in the pathogenesis of these diseases. 

## Figures and Tables

**Table 1:** First- and second-line therapies used in the treatment of neurological irAEs.

Therapy	Mechanism of Action	Contraindications	Adverse Events
FIRST-LINE TREATMENT			
Corticosteroids	Suppression of both innate and adaptive immunity, restoration of BBB disruption, reduction of the production of autoantibodies, reduction of pro-inflammatory cytokines	Acute infection Diabetes Severe osteoporosis	Hyperglycemia Osteoporosis GI ulcers Increased risk of infection
IVIG	Reduction of autoantibodies, complement inhibition, suppression of pro-inflammatory cytokines, modulation of T and B cells	IgA deficiency (allergic reactions) Severe chronic kidney disease	Acute kidney injury (tubular necrosis) Hypercoagulopathy Infusion reactions
PLEX	Filters out the plasma component of blood, thereby leading to clearance of antibodies, complement cascades, cytokines and drugs to reduce inflammation	Active infection, sepsis	Local infections Electrolyte imbalances Hypercoagulopathy
SECOND-LINE TREATMENT			
Rituximab	Anti-CD20 mAb, B-cell depleting therapy	Severe heart failure Live-attenuated vaccine Severe immunocompromised state Severe chronic infection (e.g., TB, VZV)	Hypogammaglobulinemia, Upper respiratory and UT infections
Cyclophosphamide	Alkylation of DNA leading to irreversible damage. Mainly T cells.	Pregnancy or breastfeeding (embryo-fetal toxicity) and Active UTI	Hemorrhagic cystitis Gonadal toxicity Myelosuppression Alopecia GI distress
Mycophenolate Mofetil	Purine synthesis inhibitor that leads to depletion of proliferating cells such as T and B cells	Pregnancy (neural tube defects)	Myelosuppression GI distress Liver toxicity Opportunistic infections
Azathioprine	Purine synthesis inhibitor, causes depletion of proliferating B and T cells.	Pregnancy, RA with history of alkylating agents	GI upset Cytopenia Increased risk of infections (opportunistic infections and reactivation of microorganisms) Small risk of lymphoma
Tocilizumab	Anti-IL-6R, prevents IL-6 from binding to its receptor (IL-6R) on the liver, lung	Pregnancy	Increased plasma cholesterol Increased liver enzymes (ALT, AST) Infusion-related reactions
Abatacept	Targets proinflammatory cytokines and both B and T lymphocytes	Pregnancy	Infections

irAEs: immune-related adverse events, BBB: blood–brain barrier, GI: gastrointestinal, IVIG: intravenous immunoglobulin, PLEX: plasma exchange, mAb: monoclonal antibody, TB: tuberculosis, VZV: varicella zoster virus, UT: urinary tract, UTI: urinary tract infection, RA: rheumatoid arthritis, IL-6: interleukin 6, ALT: alanine aminotransferase, AST: aspartate aminotransferase.

## Data Availability

Not applicable.
